# Editorial: Workplace Health Promotion, volume II

**DOI:** 10.3389/fpubh.2022.1102042

**Published:** 2022-12-20

**Authors:** Leah Okenwa Emegwa, Sheikh M. Alif, Danijela Gasevic

**Affiliations:** ^1^Department of Health Sciences, Swedish Red Cross University, Stockholm, Sweden; ^2^School of Public Health and Preventive Medicine, Monash University, Melbourne, VIC, Australia

**Keywords:** wellbeing, performance, psychology, mental health, social media, intervention, shift work, employee assistance program

The purpose of this Research Topic, a continuation of Workplace Health promotion Volume 1, was to understand issues connected to population health from a workplace perspective such as factors mitigating against the health of workers, barriers to occupational health promotion, and other emerging problems (1–3). The aim was also to identify enabling factors, best practices, and other crucial aspects relevant for workplace health promotion and to explore the need for a strategic approach to WHP, described as a systematic and continuous process of needs analysis, priority setting, planning, implementation, and evaluation (4, 5).

A total of 88 researchers from across the globe contributed to 13 articles in this volume. The study participants were from sectors such as health and social welfare and include doctors, medical students, social workers, and the Police, to name a few. Several of the articles explored the impact of workplace issues on mental health and wellbeing; some examples include working conditions, gossip, feelings of exclusion at the workplace, and social media usage. One study investigated the outcome of an intervention to address depression among workers. Articles in this volume also highlight the role of social support, leadership, and supervision in addressing health-related workplace challenges. Other key features include, alcohol and sick leave, shift work and insomnia, performance, and work culture, fall accidents and supervision as well as burnout and the role of sleep quality, workplace violence and shift work. Each of the articles are summarized below.

According to Chang et al. WHP performance was significantly related to workplace health culture, especially health policies, healthy climate, and peer and supervisor support. In their study, the Workplace Health Scorecard was administered to WHP representatives at each workplace, while a personal questionnaire was used to measure workplace health culture, healthy lifestyles, and health statuses at the individual level. A total of 27 enterprises and 1,732 individuals participated in the cross-sectional study, and data analyzed using a hierarchical linear model.

Using the job demands and resources (JD-R) model, Huang et al. studied the relationship between work conditions and outcomes like psychological distress among social workers in Chengdu, China. The study also investigated the mediational effects of positive affect (PA) and negative affect (NA) among the 897 participants. Results suggest that JR has a greater effect on PA and NA relative to the effects of JD on PA and NA. The authors conclude by suggesting interventions to promote PA in order to buffer against the effects of JD among social workers.

Emsing et al. using the SCL-90-R survey, aimed to find if there are differences in the mental health of two cohorts of Swedish police recruits totaling 376 from 2009 and 2020. The mental health of both cohorts was also compared to that of the general population using data from 2002. Multivariate analysis of variance (MANOVA) and bivariate analyses were conducted. Results showed that recruits from 2020 fared better, and that while some recruits scored above the Swedish patient mean, the prevalence and intensity of mental health disorders among recruits were generally low. Findings also showed that gender, educational level and relationship status were important predictors of mental health.

What is the prevalence of insomnia caused by consecutive night shifts? What night shift duration worsens insomnia the most? These were the questions which Sim et al. attempted to answer by using e.g., multivariate logistic regression to analyze night shift profiles and baseline demographics data of three hospitals between January 2015 to December 2017 (*n* = 13,025). Findings showed that the prevalence of insomnia was 38.7%, and that there was a significant relationship between consecutive night shifts and insomnia. The authors conclude that findings from the study could be a basis for developing policies and guidelines to improve the health of night shift workers.

In the study by Yan et al. the aim was to investigate the prevalence of burnout among emergency physicians and its associated factors using a nationally representative cross-sectional survey of 15,243 emergency physicians between July and September 2019 in 31 provinces across China. The prevalence of burnout was 14.9%. Associated factors were depression, shift work, workplace violence, having poor self-perceived health status and sleep quality, working in developed regions and governmental hospitals, and having an intermediate professional title. Recommendations for prevention are discussed.

Given findings from a literature review and the characteristics of fall accidents in construction, Luo et al. propose a modification of the human factor classification analysis system (HFACS) framework. They also construct a Bayesian network (BN) topology based on the dependence between human factors and organizational factors, as well as determine the sensitivity of causal factors. Findings show that the most important reason for falling accidents is unsafe on-site supervision, the BN risk assessment model suggests that the most likely causes are loopholes in site management work, lack of safety culture, insufficient safety inspections and acceptance, amongst others.

According to Kanwal and Isha social media activities among office workers in the oil and gas industry significantly moderate the effect of effort-reward imbalance on health and wellbeing and impact job rewards. The conclusions are based on exploratory factor analysis and confirmatory factor analysis of data from 424 participants using an online questionnaire. Findings showed among others, that social media activities related to work-life decreased health and wellbeing by 11%, and social media activities related to personal life negatively affected job rewards by about 55%.

Cheng et al. studied the effect of workplace gossip on employees' mental health using data collected in three waves from 222 full-time employees of a Taiwanese tourism company. Results suggest that workplace gossip is associated with employees' mental health through psychological capital, and that the relationships among workplace gossip, psychological capital, and mental health are moderated by developmental job experience.

To understand the relationship between alcohol-related problems and drinking attitudes with sick leave, Hashemi et al. linked data from the WIRUS study (Workplace Interventions preventing Risky alcohol Use and Sick leave) to company-registered sick leave data. The study included a total of 2,560 employees from 95 different work units in 9 public companies and 5 private companies in Norway. Negative binomial regression models were used, adjusted for gender, age, cohabitation status, educational attainment, work position, and employment sector. Findings show that although there were variations of 1-day, short-term, and overall sick leave days between companies than between work units within companies, neither alcohol-related problems nor drinking attitudes were associated with sick leave.

Stassen et al. conducted a systematic review to investigate the attention to principles of resistance training (RT) progression and variables of RT exercise prescription in workplace-related RT interventions. The databases searched were LIVIVO, PubMed, SPORTDiscus, and Web of Science for publications between 2000 and 2020. Results from 21 articles (18 primary studies, three protocols) revealed that interventions showed different positive effects on strength- or performance-related and/or health- or complaint-related outcomes. However, several key RT principles and variables were reported inconsistently, thus reducing reproducibility, suggesting the need for standardized RT intervention reporting in workplace-related interventions.

The study by Xu et al. aimed to investigate medical residents' levels of social support, psychological resilience, and coping style, and explore the mediating role of psychological resilience. An online questionnaire was administered to a total of 577 medical residents from China recruited *via* convenience sampling, and the data were analyzed using Pearson correlation analysis. Findings show positive correlations between social support, psychological resilience and coping style and a significant mediating effect of psychological resilience in the relationship between social support and coping styles. The importance of paying attention to the psychological status of medical residents, as well as the need for increasing enthusiasm for coping style and promoting their mental health using social support and psychological flexibility, are discussed.

Building on the stereotype content model and allostatic load theory, Frank et al. investigated whether employees with a mental illness become socially excluded at the workplace and if they have more days of sick leave. A total of 86 employees diagnosed with a mental disorder were interviewed and completed online-surveys, and Path analyses were conducted. Results show that the interview-rated severity of the mental disorder had an indirect effect on the days of sick leave, and that this was mediated by the symptomatic burden and social exclusion at the workplace. The need for organizations, especially supervisors, to be attentive for signs of exclusion and address same is discussed as important given the financial implications of employee absenteeism on organizations.

Gwain et al. conducted a pre-intervention and post-intervention study to assess an intervention which they developed to reduce depression among workers at the Outpatient Mental Health Clinic in Washington District of Columbia, United States. In order to determine the pre-intervention prevalence of depression, a survey was conducted on 43 employees using the Patient Health Questionnaire depression scale (PHQ-9). Thereafter, the WHO Healthy Workplace Model was adopted to design an instrument for determinants of depression at the workplace, followed by the development and implementation of a mHealth intervention. Finally, a post-intervention survey was conducted among the cohort, and the data was analyzed using descriptive and inferential statistics in STATA. Findings show that the prevalence of depression went from 30.2%, pre-intervention, to 12.6% post-intervention. The findings suggest that improving employee mental health can be achieved using cheap mhealth solutions.

The need for clear policys and routines, as well as increase in wellness packages offered and strategies to improve their uptake thus remains (6). In conclusion, more attention should continually be given to barriers, enabling factors and best practices for health promotion in the workplace given its strategic role for population health promotion.

## Author contributions

LO-E was responsible for project idea, design, implementation, manuscript drafting, and final version of the manuscript. DG and SA contributed to project implementation and manuscript final version. All authors contributed to the article and approved the submitted version.
