# Corrigendum: Editorial: Workplace health promotion, volume II

**DOI:** 10.3389/fpubh.2023.1152683

**Published:** 2023-02-22

**Authors:** Leah Okenwa Emegwa, Sheikh M. Alif, Danijela Gasevic

**Affiliations:** ^1^Department of Health Sciences, Swedish Red Cross University, Stockholm, Sweden; ^2^School of Public Health and Preventive Medicine, Monash University, Melbourne, VIC, Australia

**Keywords:** wellbeing, performance, psychology, mental health, social media, intervention, shift work, employee assistance program

In the published article, there was an error in the author list, and the order of the author names was erroneously presented as:

Danijela Gasevic^1^, Sheikh M. Alif^1^ and Leah Okenwa-Emegwa^2*^

^1^ School of Public Health and Preventive Medicine, Monash University, Melbourne, VIC, Australia

^2^ Department of Health Sciences, Swedish Red Cross University, Huddinge, Stockholm, Sweden

The corrected author list should be:

Leah Okenwa Emegwa^1*^, Sheikh M. Alif^1^ and Danijela Gasevic^2^

^1^ Department of Health Sciences, Swedish Red Cross University, Stockholm, Sweden

^2^ School of Public Health and Preventive Medicine, Monash University, Melbourne, VIC, Australia

The authors apologize for this error and state that this does not change the scientific conclusions of the article in any way. The original article has been updated.

